# Anti-interleukin 4 receptor α antibody for the treatment of Chinese bullous pemphigoid patients with diverse comorbidities and a 1-year follow-up: a monocentric real-world study

**DOI:** 10.3389/fimmu.2023.1165106

**Published:** 2023-07-20

**Authors:** Si-Hang Wang, Ying Shan, Si-Zhe Li, Ya-Gang Zuo

**Affiliations:** ^1^ Department of Dermatology, State Key Laboratory of Complex Severe and Rare Diseases, National Clinical Research Center for Dermatologic and Immunologic Diseases, Peking Union Medical College Hospital, Chinese Academy of Medical Sciences and Peking Union Medical College, Beijing, China; ^2^ Department of Dermatology, Hebei North University, Zhangjiakou, Hebei, China

**Keywords:** Bullous pemphigoid, dupilumab, biologics, immunosuppressant, comorbidity, severity, real-world study

## Abstract

**Background:**

Bullous pemphigoid (BP) is a common subepidermal bullous disorder that lacks adequate treatment alternatives. Dupilumab, an anti-interleukin (IL) 4 receptor α antibody blocking Th2 molecules IL-4 and 13, has been used off-label and shown to be effective in refractory BP cases.

**Methods:**

BP patients with various disease severities and comorbidities were included in this case series. All patients received dupilumab alone or in combination with immunosuppressants in a real-world setting. Complete remission (CR) was defined as the absence of pruritus symptoms and previous BP eruptions, with only hyperpigmentation patches and without newly occurring lesions for at least 4 weeks. Disease relapse was classified as the appearance of three or more new lesions within 1 month or at least one large urticarial or eczematous lesion that did not resolve within a week.

**Findings:**

Ten individuals were enrolled in this case series. Pruritus symptoms and BP eruptions improved significantly in nine patients (90%). Seven patients (70%) attained CR, including all mild-to-moderate (100%) cases and three of six (50%) severe BP cases. At the dupilumab monotherapy stage, eosinophilia was observed in two severe cases. One patient out of seven (14.3%) relapsed after 1 year of follow-up after CR.

**Conclusion:**

Treatment of BP with diverse comorbidities with anti-IL-4 receptor α antibody provides further credentials to a prospective randomized study. More impressive efficacy and safety profiles were observed in patients with mild-to-moderate disease after 1 year of follow-up. Eosinophilia may occur in patients receiving dupilumab monotherapy.

## Introduction

Bullous pemphigoid (BP) is a common autoimmune subepidermal bullous disorder that primarily affects older people, with tense bullae and pruritus as its characteristic clinical manifestations (1). Systemic and topical corticosteroids, immunosuppressants, antibiotics (minocycline or doxycycline), and nicotinamide comprise the conventional treatment for BP (1). In recent years, numerous therapeutic alternatives of biologics (such as rituximab, bertilimumab, mepolizumab, and omalizumab) targeting the signaling pathway underlying the immunopathogenesis of BP have been shown to be safe and effective in cases of therapy resistance (2–4). Although omalizumab is probably safer than rituximab, a markedly higher recurrence rate of 80% was observed after treatment was discontinued (2). As rituximab renders older patients more susceptible to severe infection, B cell depletion methods may not be an ideal therapeutic option. Consequently, additional therapeutic options with improved safety and efficacy profiles and reduced recurrence rates are required.

The importance of T helper 2 (Th2) cells and the Th2 molecule milieu in the pathogenesis of BP has been demonstrated. Dupilumab is an interleukin (IL)-4 receptor α antagonist that blocks both the IL-4 and IL-13 pathways and has demonstrated efficacy in atopic dermatitis (AD) treatment (5). In 2017, the U.S. Food and Drug Administration approved dupilumab for treating moderate-to-severe AD. In the past 6 years, numerous cases reported outstanding outcomes in numerous BP patients treated with dupilumab. Importantly, dupilumab treatment largely accelerated a tapering course of concomitant immunosuppressive therapies with a lower recurrence rate in a 32-week follow-up and has achieved disease clearance in a shorter period than conventional immunosuppressive therapy alone, and no dupilumab-related adverse events have been recorded (6). Herein, we describe therapeutic strategies in ten Chinese BP patients with diverse comorbidities and different severities treated with dupilumab in an unstrained real-world setting during the 1-year follow-up period and provide an overview of the current literature.

## Methods

Patients from the Department of Dermatology, Peking Union Medical College Hospital (PUMCH), were recruited in the case series. All patients were diagnosed with BP using at least three of the following methods: (i) hematoxylin and eosin staining demonstrating subepidermal blisters and eosinophil infiltration; (ii) direct immunofluorescence staining showing a linear deposition of IgG/IgM or complements at the basement membrane zone (BMZ); (iii) serum detection of IgG autoantibodies against BP180 (BPAG2) via an enzyme-linked immunosorbent assay; and (iv) indirect immunofluorescence showing circulating IgG antibodies binding to the BMZ. All patients in our case series have an average follow-up duration of more than 1 year.

Patients who had taken medications (such as furosemide, spironolactone, amiodarone, gliptins, anti-PD-1, and anti-PD-L1) (7) known to cause or exacerbate BP were excluded in this case series. Additionally, precluded patients were those with internal malignancies, epidermolysis bullosa acquisita, or other blister diseases. This investigation was approved by the ethics committee of PUMCH (ethics document number: JS-3132) and was conducted in accordance with the Declaration of Helsinki. All patients or their legal guardians gave their informed written consent.

We evaluated the clinical response and adverse effects of dupilumab in ten Chinese BP patients with varying comorbidities and severities. An initial dose of 600 mg was administered subcutaneously (SC), followed by 300 mg SC every 2 weeks in cases 2-9, and 300 mg every week in case 10 because of a serious disease condition. Owing to the severity of the disease and a more stable financial situation, the patient in case 1 initially received three doses of 600 mg preload dose every 2 weeks, followed by 300 mg every 2 weeks. Furthermore, nine of ten (90%) patients also received immunosuppressive therapy, such as a tapering course of methylprednisolone and *Tripterygium wilfordii* Hook F (TwHF), during dupilumab treatment (**Table 1**). TwHF is a traditional Chinese medicine used to treat rheumatoid arthritis, Crohn’s disease, and cutaneous inflammatory diseases, such as chronic urticaria and BP, due to its anti-inflammatory and immunomodulatory properties (9). We previously reported ten BP patients treated with TwHF and demonstrated a favorable safety profile and good tolerance in mild-to-moderate BP patients. TwHF was used as a corticosteroid-sparing regimen and maintenance therapy after dupilumab discontinuation. TwHF is well tolerated and efficacious at doses between 30 and 60 mg per day in patients with mild or moderate BP, but this dosage is insufficient for severe BP cases (10). The clinical efficacy of dupilumab was determined by the improvement of BP lesions (tense bullae, erythematous patches, or urticarial plaques) and pruritus symptoms. Complete remission (CR) was defined as the complete relief of pruritus symptoms and clearance of previously recognized BP eruptions, with only hyperpigmentation patches and without newly occurring lesions for at least 4 weeks. The average Bullous Pemphigoid Disease Area Index (BPDAI) was used for evaluating skin conditions and defining disease severity at the time of dupilumab initiation. BPDAI cutoff values distinguishing mild (<20), moderate (20–57), and severe BP (>57) were used as an objective measure of BP activity (8). Relapse was classified as the appearance of three or more new lesions within 1 month or at least one large urticarial or eczematous lesion that did not resolve within a week.

**Table 1:** Demographics of BP patients treated with dupilumab.

Case	Age/sex	Disease course (before dupilumab initiation)	Comorbidities	BPDAI/BPDAI pruritus component/disease severity* (before dupilumab initiation)	Prior treatment	Concomitant therapies with dupilumab	Dupilumab treatment durations (weeks)	Outcome of dupilumab (time to achieving CR)	Relapse after discontinuation of dupilumab (from CR)
1	67/M	4 months	Hypoproteinemia, hyperglycemia	134/30 Severe	MP, minocycline	MP tapered from 16 mg to 4 mg daily, TwHF	18	CR (14 weeks)	Yes (21 weeks)
2	84/F	2.5 years	Serious osteoporosis	16/10 Mild	MP	Tapering course of MP, off in 2 months	48	CR (6 weeks)	No
3	81/M	5 months	Diabetes, cerebral infarction, hypertension	42/12 Moderate	Minocycline, niacinamide, TwHF, MP, topical corticosteroids, antihistamines	MP tapered from 20 mg to 4 mg daily, TwHF	24	CR (7 weeks)	No
4	68/M	1 year	Pneumocystis pneumonia and cytomegalovirus infection	45/12 Moderate	MP	MP tapered from 20 mg to 8 mg/day	48	CR (8 weeks)	No
5	71/M	2 months	Diabetes, cerebral infarction, hypertension	34/10 Moderate	TwHF, topical corticosteroids, antihistamines	TwHF	38	CR (7 weeks)	No
6	98/M	2 months	Hypertension, coronary heart disease	62/27 Severe	Minocycline, niacinamide, topical corticosteroids	None	14	CR (10 weeks)	No
7	35/M	1.5 months	Allergic rhinitis	106/20 Severe	MP	Tapering course of MP from initial 40 mg/day to 20 mg/day after three injections of dupilumab	48	CR (6 weeks)	No
8	60/F	6 years	ONFH, herpes zoster	99/26 Severe	MP, IVIG	TwHF, betamethasone 7 mg after four courses of dupilumab, topical corticosteroids	44	Pruritus and bullae improved	No (6 months after dupilumab discontinuation)
9	74/F	2 months	Cardiac insufficiency, kidney insufficiency, hyperkalemia	150/27 Severe	Topical corticosteroids, antihistamines	MP 40 mg daily after two injections of dupilumab, tapered by 20 mg 4 days later. TwHF (60 mg daily) after three injections of dupilumab, MP (16 mg daily) after three injections of dupilumab, 32 mg daily after four injections of dupilumab	6	No improvement in bullae and pruritus	Not applicable
10	89/M	9 months	Hypertension, hyperlipidemia, atrial fibrillation, deep vein thrombosis, Alzheimer’s disease	130/25 Severe	Topical corticosteroids, antihistamines, minocycline	TwHF, minocycline (100 mg daily) after 14 weeks of dupilumab initiation	30	Pruritus and bullae improved	Not applicable

BP, bullous pemphigoid; F, female; M, male; TwHF, Tripterygium wilfordii Hook F; MP, methylprednisolone; IVIG, intravenous immunoglobulin; ONFH, osteonecrosis of the femoral head; BPDAI, The Bullous Pemphigoid Disease Area Index (total maximum score of 372); BPDAI pruritus component (total maximum score of 30).

*BPDAI cutoff values of 20–57 were used to define mild, moderate, and severe BP (8).

## Results

**Table 1** details the demographics of patients receiving dupilumab therapy. Ten patients (seven males) who received dupilumab therapy in our department and had varying comorbidities and disease severities were enlisted. The enrolled patients had previously received a variety of immunosuppressive therapies for BP, including systemic and topical corticosteroids, TwHF, intravenous immunoglobulin, minocycline, and nicotinamide. These patients proceeded with dupilumab because of refractory BP, contraindications to conventional therapies, or a poor general condition. Before dupilumab started, the average BPDAI and BPDAI pruritus components were 81.8 (range 16–150) and 19.9 (range 10–30), respectively. Six patients (cases 1, 6, 7, 8, 9, and 10), three patients (cases 3, 4, and 5), and one patient (case 2) were respectively defined as severe, moderate, and mild BP at the time of dupilumab introduction. The average duration of BP before initiating dupilumab and treatment with dupilumab was 14.0 months (range 1.5–72 months) and 28.2 weeks (range 6–30 weeks), respectively.

Nine patients (90%) receiving dupilumab therapy showed notable improvements in their pruritus symptoms and BP eruptions. Seven patients (70% of the total) obtained CR. On average, it took 8.3 weeks (58 days) for seven patients to achieve CR. In our case series, 100% of mild-to-moderate cases attained CR. Of the severe BP patients (cases 1, 6, 7, 8, 9, and 10 in which BPDAI exceeded 57) (8), three patients (50%) achieved CR (cases 1, 6, and 7), two patients experienced partial remission (case 8 and 10), and one patient did not experience any improvement (case 9). One patient (case 1) out of seven (14.3%) relapsed after 1 year of follow-up after CR.

**Figure 1:**
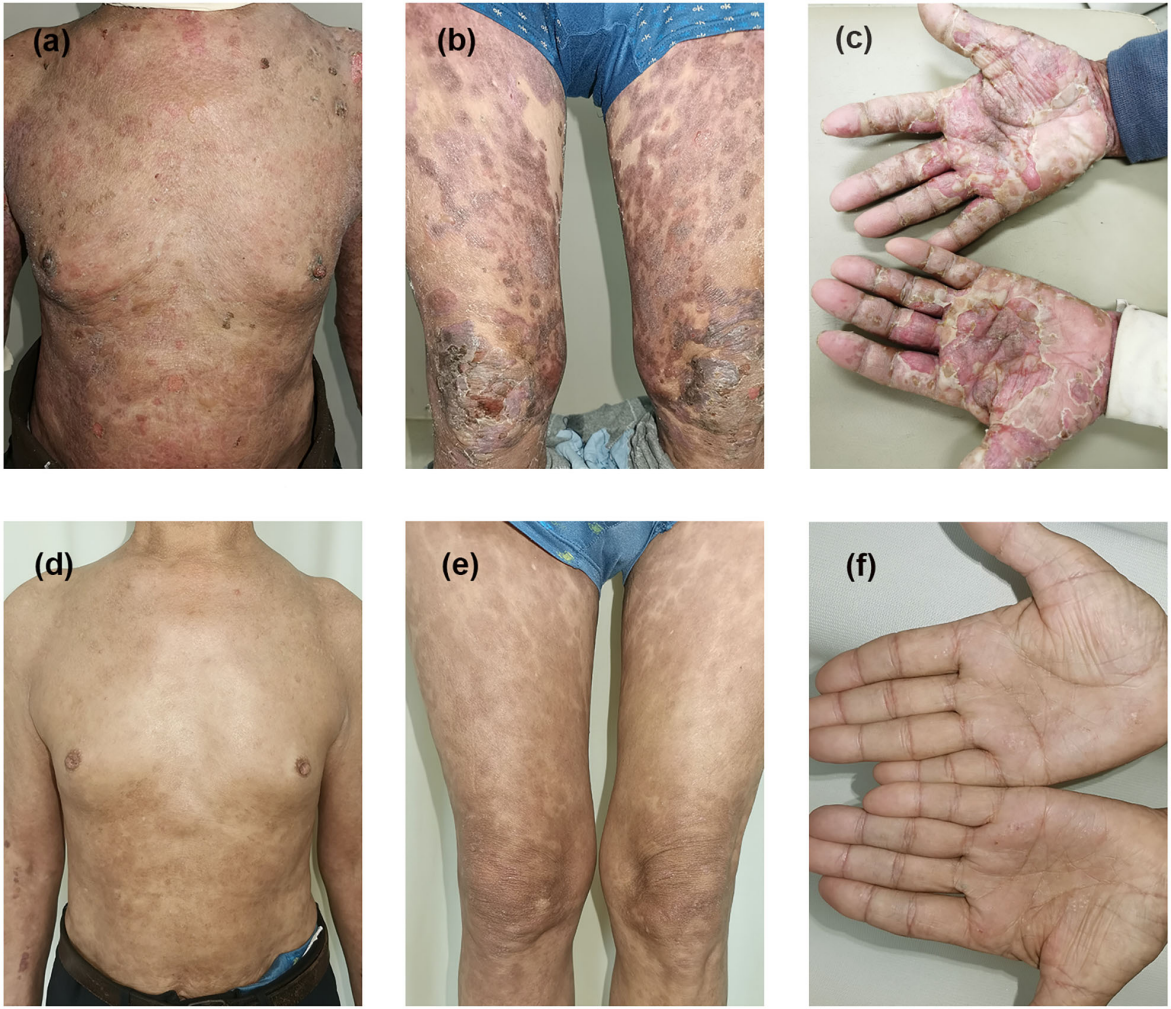
Clinical presentation before and after therapy in case 1. Erythematous patches and tense bullae appear on the trunk, extremities, and hands before treatment **(A–C)**. Bullae and crusting disappeared with hyperpigmentation **(D–F)** after 14 weeks of treatment with dupilumab combined with immunosuppressive therapy.

In case 1, the patient initially received three courses of a higher dose of 600 mg SC every 2 weeks, followed by 300 mg SC every 2 weeks. In the interim, 40 mg of TwHF was also administered. The fact that the daily dose of methylprednisolone in case 1 was 16 mg, which is far below the recommended prescribing regimen of 0.75–1 mg/kg for severe patients (1), suggested that systemic steroids played a limited role in the case. After seven courses of dupilumab treatment, he eventually achieved CR; thus, dupilumab was discontinued after the ninth injection. The patient maintained CR with a low dose of methylprednisolone (4 mg per day) in conjunction with 20 mg of TwHF per day (**Figure 1**). We observed a substantial reduction in eosinophils (from 41.7% before treatment to normal after five injections). Additionally, sera autoantibody titers of anti-BP180 became negative after five doses of dupilumab. Twenty-one weeks after the withdrawal of dupilumab, he experienced a severe flare-up of BP. Treatment with 600 mg of dupilumab once followed by 300 mg every 2 weeks, 16 mg of methylprednisolone, and 60 mg of TwHF daily was reinitiated. The patient reported CR 8 weeks after resuming treatment.

A 98-year-old male patient with intolerable pruritus for at least 1 month presented to our department in case 6. He suffered from hypertension and coronary heart disease in an inferior general condition. BP symptoms improved after treatment with topical corticosteroids, minocycline, and niacinamide for 3 weeks. However, he swiftly developed new blisters and experienced intractable itching. Owing to contraindications with oral corticosteroids and extremely old age, dupilumab monotherapy was introduced. Prior to dupilumab administration, the eosinophil count and anti-BP180 antibody concentration were 5.9% and 16 U/ml, respectively. However, there were no laboratory results after treatment because he was unable to visit the hospital during the COVID-19 pandemic. We made contact via the PUMCH online communication system during the follow-up period. Two days after the first injection, his pruritus significantly improved, and he could fall asleep soundly. After five courses of dupilumab monotherapy, he reported CR and no new lesions. Overall, the patient received a total of seven courses of dupilumab monotherapy and was completely symptom free. During the 1-year follow-up period, there were no reports of disease flare-ups.

In case 7, a younger patient initially received 40 mg of methylprednisolone per day, but more than 50 new blisters developed after 3 days of treatment, and he suffered from severe pruritus. The initial loading dose of 600 mg of dupilumab by injection was prescribed. He reported a significant improvement in previously identified skin lesions and pruritus symptoms the next day. Following two injections of dupilumab, he was nearly free of blisters and had smaller erythematous regions. Additionally, a substantial decrease in eosinophil counts was observed (from 20% before therapy to 0.1% after the first course of dupilumab). The patient reported no new blister formation and complete symptom alleviation after three injections of dupilumab. Anti-BP180 antibody titers decreased from over 150 U/L to 113 U/L after 12 weeks. Additionally, dupilumab markedly facilitated methylprednisolone tapering (from an initial dose of 40 mg/day to 10 mg/day in 6 months).

Case 8 patient reported a reduction in the edema of the lower limbs and feet after the first injection of dupilumab. However, she continued to develop new blisters on the right upper extremity. The percentage of eosinophils significantly increased from 38.8% before treatment to 65.2% and 67.7% 1 and 2 weeks after the first injection, respectively. She experienced intense pruritus and impatience. We introduced the immunosuppressant TwHF at a dosage of 40 mg daily after two courses of dupilumab. Additionally, topical corticosteroids were applied to her newly formed blisters. The patient reported a marked reduction in skin lesion counts and pruritus after TwHF introduction. Additionally, eosinophil levels decreased to 31%. However, 2 days after the fourth injection of dupilumab, she described a relapse characterized by the appearance of new erythema on her face. A compound betamethasone injection (containing 7 mg of betamethasone) was prescribed. After 4 days of betamethasone injections, her skin lesions improved significantly, and her eosinophil count returned to normal levels. Until the present time, the patient has reported CR of skin lesions and pruritus with a normal eosinophil count. We did not rule out the roles of TwHF and betamethasone and classified the patient as being in partial remission even though the patient ultimately attained satisfactory results. During the 44 weeks of treatment with dupilumab plus TwHF and topical corticosteroid, we observed a significant decrease in total IgE (from 1,090 IU/ml after three injections to 33.9 IU/ml) and anti-BP180 (from 115 U/ml before dupilumab initiation to 20 U/ml). After 44 weeks of combination treatment, we chose to discontinue dupilumab. At her most recent clinic visit (six months subsequent to CR), she was still maintaining CR with 40 mg of TwHF daily. No recurrence of the disease was reported. It was noteworthy that the patient had herpes zoster 3 months previously, and treatment with dupilumab did not result in a recurrence of the herpes virus.

Case 9 showed a modest improvement in pruritus and bullae after two courses of dupilumab. However, the eosinophil count was markedly increased to 38.7%, 37.6%, and 42.4% 3 days, 1 week, and 2 weeks after the first injection, respectively. We had to reinitiate conventional systemic immunosuppressive therapies. Intravenous methylprednisolone (40 mg daily) was administered 1 week after two injections of dupilumab. Four days later, she experienced a perceptible reduction in itching, and no new blisters appeared. We tapered methylprednisolone to 20 mg daily while prescribing 60 mg of TwHF daily. After three administrations of dupilumab, methylprednisolone dosage was reduced by 16 mg daily. However, the patient reported a relapse of BP four days after methylprednisolone tapering. She suffered intolerable pruritus and the rapid development of over 30 new lesions. The daily dose of methylprednisolone was increased to 32 mg so that she ultimately achieved disease control. While receiving anti-IL-4 receptor therapy, cardiac insufficiency, kidney insufficiency, and hyperkalemia did not worsen in this case. This patient was classified as having no improvement because disease control was achieved by increasing the dosage of conventional systemic immunosuppressive therapies. In cases 8 and 9, the eosinophil count was substantially elevated following dupilumab monotherapy. In both patients, parasite infection, allergic diseases, and eosinophilic leukemia were ruled out. We proposed that eosinophilia following the initiation of dupilumab could be an adverse event in both cases.

Case 10 was referred to our dermatology department due to resistance to traditional BP treatment. We implemented a systemic combination therapy consisting of 300 mg of dupilumab SC every 2 weeks and 40 mg of TwHF daily. After 2 weeks of combination therapy, the patient reported a persistent increase in the number of bullae (5, 6). The injection interval was increased to a weekly administration of 300 mg. Then, he described a reduction in lesion counts and alleviation of pruritus over the following weeks, despite the absence of disease control. Minocycline (100 mg daily) was prescribed after seven courses of combination therapy, and he finally achieved CR; thus, the injection interval was reduced to 300 mg every 2 weeks.

In the case series, four mild-to-moderate patients (BPDAI not exceeding 57) (8) attained CR with dupilumab in conjunction with a tapering course of methylprednisolone (cases 2–4) and TwHF (cases 3 and 5). The treatment with dupilumab was well tolerated even though all of the patients were over the age of 60 and had severe serious complications, such as osteoporosis, diabetes, hypertension, or cerebral infarction. Anti-IL-4 receptor α therapy did not cause a recurrence of pneumocystis pneumonia in case 4. During the 1-year follow-up period following CR, there were no reports of disease recurrence in mild-to-moderate patients. No adverse events related to dupilumab were documented in cases 1–7 and 10.

## Discussion and overview of literature

**Figure 2:**
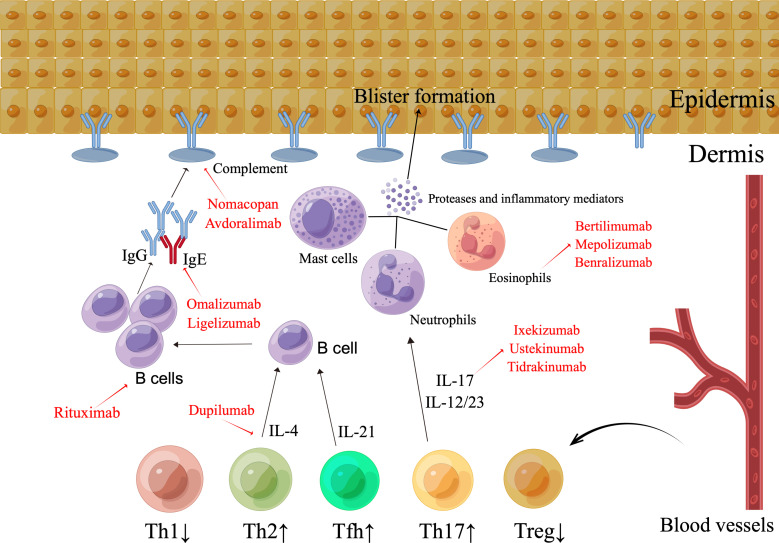
The pathogenesis and emerging therapeutic strategies in bullous pemphigoid. The existence of IgG antibodies and the complement element C3, which attacks the BMZ, characterizes BP. Immune complex production triggers the activation of complement, which results in the migration of eosinophils, mast cells, and neutrophils, as well as the secretion of proteases and inflammatory markers, thereby inducing dermal-epidermal split. The pattern of distribution of the subsets of T cells is atypical. The Th2, Tfh, and Th17 groupings are elevated, whereas the Treg cell count is diminished. By means of the release of IL-17, aberrant Th17 cell types enhance the pro-inflammatory immune reaction, stimulate neutrophils, magnify the inflammatory process, and promote tissue injury. Th2 cells and Th2 cell-released IL-4 stimulates B cell growth, antibody synthesis, and class-switching of immunoglobulins. The improper functioning of Treg cells promotes the activation of autoreactive CD4+ T lymphocytes and the creation of autoantibodies. Enhanced Tfh proliferation facilitates the production of autoantibodies in BP by B cells. Possible targets range from CD20+ lymphocytes with rituximab to the Th2 axis with dupilumab, and omalizumab or the IL-17/IL-23 axis, as well as the inhibition of particular complement or inflammatory mediators.

BP, the most prevalent autoantibody-mediated cutaneous blistering disorder, is characterized by pruritic vesicles or bullae and primarily affects older people. Pathogenic B and T cells, autoantibodies, and inflammatory cytokines/chemokines are significant in the characteristic pathology of BP (1) (**Figure 2**). Almost all BP patients have circulating IgG autoantibodies against the non-collagenous 16A (NC16A) domain, the immunodominant region of BP180, which correlates with the severity of this disorder (1, 11, 12). Importantly, these autoantibodies are synthesized by autoreactive B cells, to which type 2 T helper (Th2) and T follicular helper cells (Tfh) increase antibody production and humoral immunity via the secretion of numerous inflammatory cytokines/chemokines (13). Th2 cells and the milieu of Th2 molecules have been shown to play a vital part in BP pathogenesis (14–16).

A potent inhibitor of Th2-related chemokines (such as CCL17, CCL18, CCL22, and CCL26) in AD patients, dupilumab, an IL-4 receptor antagonist blocking both IL-4 and IL-13 signaling pathways, significantly improves the molecular signature of AD and exhibits a satisfactory efficacy and safety profile (5). Moreover, various studies have reported its efficacy in therapy-refractory dermatological conditions, such as prurigo nodularis, chronic pruritus, chronic hand eczema, allergic contact dermatitis, urticaria, alopecia areata, and BP (17). Dupilumab has been successfully used to treat a large number of BP cases in recent years, either as a monotherapy or in combination with anti-IgE therapy or immunosuppressants. A multicenter case series and a case-control study have suggested a high disease clearance rate in 12 of 13 (92%) patients and a significantly reduced time of new blister formation in BP patients (6, 18). Since then, several case reports have shown dupilumab to be an effective treatment for different subtypes of pemphigoid, including adolescent BP, eczema-like BP, IgA BP, anti-PD-1/PD-L1-induced BP, vesicular pemphigoid, and pemphigoid nodularis (19–32). Dupilumab was suggested in the recently created European guideline for BP management released by the European Academy of Dermatology and Venereology. It is currently undergoing a phase 3 clinical trial (NCT04206553).

Our current case series indicates that anti-IL-4 receptor α therapy in combination with immunosuppressive medications is well tolerated even in older BP patients with severe cardiovascular, metabolic, endocrine, infectious, and renal complications or other poor general conditions. Moreover, all mild and moderate patients in our case series achieved CR, and no disease flare-up was noted during the 1-year follow-up period, pointing to excellent efficacy profiles and a low recurrence rate in mild-to-moderate patients receiving dupilumab combined with methylprednisolone and/or TwHF. In these patients, dupilumab therapy also greatly aided the tapering of immunosuppressive medication. In comparison with rituximab (average of 148 days to CR), seven patients in this case series experienced CR 58 days earlier on average, which suggests early effectiveness in dupilumab-treated individuals (33). A very encouraging finding was that only one of ten (10%) patients in our case series experienced a disease flare-up during the 1-year follow-up period; other biologics, such as omalizumab, have a higher recurrence rate of 80%. Dupilumab monotherapy was initially used for two cases of severe BP (cases 8 and 9) in the case series, but only modest success was observed. To control the condition, traditional systemic immunosuppressive treatments, such as TwHF and methylprednisolone, are required. After the effectiveness of conventional immunosuppressive therapies, dupilumab treatment conduced to the tapering course of systemic immunosuppressive therapies in case 8. However, after three dupilumab injections, the dupilumab therapy was stopped as it was ineffective in case 9. High doses of traditional immunosuppressive medications must be restarted. In case 10, an increased injection interval was well tolerated and facilitated pruritus alleviation, while disease control was absent. In eosinophilic esophagitis, a weekly dose of 300 mg of dupilumab contributed to the histologic outcomes and symptom alleviation (34). Therefore, it might be considered in very rare cases of BP, and clinical investigations comparing different dosages are warranted. In older patients, minocycline may be an optional and secure concurrent therapy. We anticipated that more concurrent immunosuppressive regimens would be necessary among severe patients to attain disease control. Overall, dupilumab has demonstrated an extraordinary safety and inspiring efficacy profile with a lower recurrence rate in BP patients with various comorbidities in poor general conditions.

When conventional treatments had not yet been prescribed, we discovered a substantial rise of eosinophil counts in two patients with severe BP receiving dupilumab monotherapy. Eosinophilia was documented as an adverse event in 52 patients (4.1%) who received dupilumab for asthma treatment, and 59 patients (9.0%) for AD and mainly were laboratory results without clinical symptoms (only 0.2% of patients receiving asthma treatment were associated with eosinophil-related severe clinical adverse results [aggravation of hypereosinophilia and chronic eosinophilic pneumonia]) (35, 36). It was conjectured that initial dupilumab blocks both IL-4 and IL-13 functions but not IL-5, leading to a momentary release of eosinophils from bone marrow (35). In case 8, the eosinophil count increased from 38.8% before the start of dupilumab monotherapy to 65.2% and 67.2% after 1 and 2 weeks, respectively. However, a reduction of the edema on her lower extremities with a few new blisters on the right upper extremity was noticed after the first injection. For case 9, we were unable to obtain baseline eosinophil levels because she was late for the blood collection. After 3 days and 1 and 2 weeks after the first injection, her eosinophil count was 38.7%, 37.6%, and 42.4%, respectively. Additionally, the patient exhibited a very slight improvement in pruritus and bullae. When eosinophils returned to normal levels after introducing conventional immunosuppressive therapies, injection with dupilumab continually did not result in eosinophilia in both cases. However, eosinophilia, was not observed in other patients. Concomitant immunosuppressants, according to our hypothesis, could lower eosinophil numbers. As eosinophilia is a common laboratory manifestation in the active phase of BP patients, uncontrolled BP disease that causes eosinophilia cannot be completely ruled out.

BP is currently a therapeutic challenge, as the validated treatments are corticosteroids and corticosteroid-sparing immunosuppressants, the efficacy of which is counterbalanced by their low safety profile with long-term use. The most recent findings on the pathogenesis of BP have provided an impetus for additional research aimed at identifying novel target treatments for refractory cases, with the intent of ensuring long-term effective and safe treatments for BP patients. Possible targets range from CD20+ lymphocytes with rituximab to the Th2 axis with dupilumab, and omalizumab or the IL-17/IL-23 axis, as well as the inhibition of particular complement or inflammatory mediators. The evidence of these new therapeutic targets and specific investigational compounds is shown in **Table 2** and **Figure 2**.

**Table 2:** Novel Biologics in the treatment of BP.

Novel biologics	Patients number/therapeutic targets	Dose regime	Concomitant therapies	Efficacy	Adverse reactions	References
**Rituximab**	13	500 mg weekly for 4 weeks	Pred	>90%	Infections	(37)
	12	375 mg/m^2^ weekly for 4 weeks	IVIG	100%	None	(38)
	28	500 or 1,000 mg on day 1 and day 15	None	67.9%	None	(39)
	62	Initial dose of 375 mg/m^2^ every 1–4 weeks to 500 mg weekly for 2 weeks	None	85%	Infections, anemia, neutropenia, syndrome of inappropriate antidiuretic hormone secretion (SIADH), drug fever, acute pruritus, peripheral arterial occlusive disease and tachycardia.	(2)
	20	375 mg/m^2^ weekly for 4 weeks or 1,000 mg on day 1 and day 15	Pred, MMF, AZA, MTX	75%	infections	(40)
	38	375 mg/m^2^ weekly for 4 weeks or 1,000 mg on day 1 and day 15	Pred	76%	None	(41)
**Omalizumab**	1	300 mg every 2 weeks	NA	Disease control	NA	(42)
	1	300 mg every 2 and 4 weeks	NA	Disease control	NA	(43)
	1	300 mg every 4 to 8 weeks	AZA, pred	Complete remission	None	(44)
	6	375 mg every 2 weeks and 300 mg every 8 weeks	Pred, AZA	2 complete remission, 3 symptom-free, 1 terminated due to intercurrent disease	COPD exacerbation due to termination of Pred, epigastric pain, mild elevation of liver enzymes	(45)
	2	300 mg every 3 to 4 weeks; 300 mg every three weeks	Pred	Free of pruritus and few blisters	None	(3)
	1	300mg every 4 weeks	Pred	Complete remission	Thrombocytopenia	(46)
	11	300 mg every 2 to 4 weeks and 300 mg every 8 weeks	Pred, AZA, MP, TCS	6 Complete remission, 1 partial remission, 4 not available	Elevation of liver enzymes, thrombocytopenia, two myocardial infarctions not directly due to omalizumab	(47)
	1	300 mg monthly	TCS	Complete remission	None	(48)
Complement system inhibitors						
**Nomacopan**	9/complement 5a and leukotriene B4 inhibitor	90 mg day 1 and 30 mg daily until day 42	None	7 of 9 patients with a decreased BPDAI score	NA	(49)
**Avdoralimab**	40/anti-C5aR1 antibody	3 subcutaneous injections of avdoralimab every week for 12 weeks	TCS	ongoing	ongoing	NCT04563923
**Bertilimumab**	11/eotaxin-1 inhibitor	Intravenous (10 mg/kg), 3 courses biweekly	Pred	81% reduction in disease severity	NA	NCT02226146
**IL-5 inhibitors**						
**Mepolizumab**	30/IL-5 inhibitor	750 mg every 4 weeks for 4 months	Pred	No superiority of mepolizumab versus placebo	None	(50)
**Benralizumab**	120/IL-5R α-subunit inhibitor	Benralizumab subcutaneous loading dose followed by repeat dosing	Pred	ongoing	ongoing	NCT04612790
**IL-17 and IL-23 inhibitors**						
**Ixekizumab**	4/IL-17A inhibitors	160 mg subcutaneously at day 0, 80 mg at weeks 2, 4, 6, 8, 10, and 12	None	Lack of benefit	NA	NCT03099538
**Ustekinumab**	18/IL-12/23 p40 subunit inhibitor	90 mg subcutaneously at day 0 and week 4 and 16	TCS	Ongoing	Ongoing	NCT04117932
**Tildrakinumab**	16/IL-23 inhibitor	160 mg at week 0, 4, and 16	None	NA	NA	NCT04465292
**Ligelizumab**	20/anti-IgE monoclonal antibody	240 mg subcutaneously every 2 weeks	None	Predefined efficacy was not met	NA	NCT01688882
**AC-203**	10/IL-1β modulator	Ointment applied twice daily	None	Terminated with partial enrollment completed	NA	NCT03286582

BP, bullous pemphigoid; IL, interleukin, Pred, prednisone; IVIG, intravenous immunoglobulin; TCS, topical corticosteroid; NA, not available; TwHF, Tripterygium wilfordii Hook F; MP, methylprednisolone; BPDAI, The Bullous Pemphigoid Disease Area Index; MMF, mycophenolate mofetil; AZA, azathioprine; MTX, methotrexine.

Eosinophil accumulation and eosinophilic spongiosis are prevalent features of BP, and numerous published data support the essential pathogenic function of eosinophils in BP. In the presence of eosinophil-related cytokines and chemokines, IL-5-activated eosinophils migrate to the BMZ, resulting in derma-epidermal separation (51). Experimental treatments targeting eosinophilic-related pathways in BP, including benralizumab and mepolizumab, are currently being tested in stage 2 and 3 clinical trials (50) (**Table 2**). The role of eotaxin in recruiting activated eosinophils at the BMZ during BP supports its role as a therapeutic target, and the results of a crucial phase 2 clinical study conducted on nine patients with moderate-to-severe BP treated with bertilimumab render this molecule a promising target (**Table 2**).

Several lines of research point to an essential role of the IL-17/IL-23 pathway in the pathogenesis of BP. Increased concentrations of IL-17A+ lymphocytes with CD4 expression have been identified in the peripheral blood of patients with BP, and genes encoding the IL-17/23 pathways have been found to be amplified among individuals with BP (52, 53). Given the crucial function of the IL-17/IL-23 axis in the development and progression of BP, current investigations on antagonists of the IL-17/IL-23 axis in BP are viewed with great anticipation (**Table 2**).

Preliminary evidence has demonstrated a prolonged period of remission, steroid-sparing activity, and a satisfactory safety record in patients with severe BP treated with rituximab. Moreover, the evidence supporting the use of omalizumab as an additional agent in the treatment of BP is accumulating. Omalizumab and rituximab are currently administered as additional therapeutic modalities in BP treatment (7). Although the published literature do not favor one therapeutic option over the other, contraindications to rituximab, such as severe infection, immunosuppression, and serious cardiac insufficiency, probably suggest an undesirable safety profile, especially in older people who tended to have numerous systemic complications or were in a poor general condition. Moreover, high recurrence rates documented in omalizumab prevent its use in clinical practice.

Limitations exist in this study. We cannot conclude the appropriate dosage and the interval of dupilumab for BP treatment because of the small sample size, short follow-up period, lack of a control group, single-center research, and a retrospective study design. Instead of dupilumab monotherapy, the majority of BP patients received immunosuppressive treatments concurrently. Further clinical trials are needed to deal with these unresolved issues.

Our current case series on anti-IL-4 receptor α therapy in treating BP combined with immunosuppressants provides further credentials to a prospective randomized study. A deep understanding of Th2 inflammation and anti-Th2 therapy may yield the clinical development of better-targeted therapies in BP patients.

## Data availability statement

The raw data supporting the conclusions of this article will be made available by the authors, without undue reservation.

## Ethics statement

This study was approved by the ethics committee of Peking Union Medical College Hospital (ethics document number: JS-3132). The patients/participants provided their written informed consent to participate in this study.

## Author contributions

S-HW wrote the manuscript with significant contributions from Y-GZ. S-ZL and YS assessed the Bullous Pemphigoid Disease Area Index. Y-GZ collected clinical pictures and revised the article critically. All authors contributed to the article and approved the submitted version.
